# Comparative Analysis of Ultrasound-Guided Erector Spinae Plane Block and Retro-laminar Block on Postoperative Pain Following Upper Abdominal Laparoscopic Surgery

**DOI:** 10.5812/aapm-158242

**Published:** 2025-05-26

**Authors:** Poupak Rahimzadeh, Seyed Hamid Reza Faiz, Mahmood-Reza Alebouyeh, Faranak Rokhtabnak, Reza Farahmand Rad, Shima Movaseghi

**Affiliations:** 1 Pain Research Center, Department of Anesthesiology and pain medicine, School of Medicine, Iran University of Medical Sciences, Tehran, Iran; 2 Minimally Invasive Surgery Research Center, Department of Anesthesiology and pain medicine, School of Medicine, Iran University of Medicine Sciences, Tehran, Iran; 3 Department of Anesthesiology and pain medicine, School of Medicine, Iran University of Medical Sciences, Tehran, Iran; 4 Department of Anesthesiology, School of Medicine, Iran University of Medical Sciences, Tehran, Iran; 5 Department of Anesthesiology and Pain Medicine, School of medicine, Iran University of Medical Sciences, Tehran, Iran; 6 School of Medicine, Iran University of Medical Sciences, Tehran, Iran

**Keywords:** Retrolaminar Block, Erectorr Spinae Plane Block, Pain Management, Ultrasound, Laparoscopic Surgery

## Abstract

**Background:**

Postoperative pain following laparoscopic surgeries, such as laparoscopic cholecystectomy, can be severe. Despite various analgesic methods, high doses of narcotics are often required, leading to complications such as dizziness, respiratory disorders, and postoperative nausea and vomiting (PONV).

**Objectives:**

The present study aimed to evaluate the efficacy of two novel analgesic methods, the erector spinae plane block (ESPB) and the retrolaminar block (RLB), performed under ultrasound guidance, in managing pain after upper abdominal laparoscopic surgeries.

**Methods:**

In this clinical trial, candidates for elective upper abdominal laparoscopic surgeries were randomly assigned to two groups (40 patients in the ESPB group and 40 in the RLB group). To manage preoperative pain, one group received an ESPB block under ultrasound guidance on the surgical side, while the other group received a RLB. Both groups were equipped with a patient-controlled intravenous analgesia (PCIA) pump containing fentanyl. The analgesic used in both blocks was 0.1% ropivacaine (20 cc) on the surgical side. Patients’ pain intensity [based on the Numeric Rating Scale (NRS)], need for additional narcotics, satisfaction, and sedation scores were recorded and analyzed at various time points post-surgery.

**Results:**

There was no statistically significant difference in the demographic and baseline characteristics between the two groups. However, the average NRS score was significantly lower in the RLB group at all time points post-surgery, except immediately after surgery (P < 0.001). Patient satisfaction was higher in the RLB group at 20 minutes, 2 hours, 4 hours, and 6 hours post-surgery (P < 0.05). The RLB group also required fewer narcotics, indicating that the RLB is more effective in managing acute postoperative pain.

**Conclusions:**

The RLB is more effective than the ESPB in reducing post-laparoscopic cholecystectomy pain. It also decreases narcotic consumption and associated complications. Therefore, it is recommended as a cost-effective method for managing acute pain after laparoscopic cholecystectomy.

## 1. Background

Postoperative pain following laparoscopic surgeries, such as laparoscopic cholecystectomy, can be significant, impacting patient recovery (1). Laparoscopic cholecystectomy is the gold standard for treating gallbladder diseases (2). Pain from laparoscopic surgery may reduce respiratory function, delay ambulation, and extend hospital stays (3). Traditional pain management often relies on high doses of narcotics, which can cause complications such as dizziness, respiratory depression, and postoperative nausea and vomiting (PONV) (4). These challenges have driven the exploration of alternative analgesic techniques to improve patient outcomes (5). Ultrasound-guided nerve blocks have become integral to multimodal postoperative analgesia due to their safety, precision, and efficacy (6). Anesthesiologists prioritize techniques that are minimally invasive, quick to perform, and provide robust pain relief (7). Among these, the erector spinae plane block (ESPB) and retrolaminar block (RLB) have emerged as promising options for managing postoperative pain in upper abdominal surgeries (8-10). These techniques leverage ultrasound technology to enhance accuracy and effectiveness, offering potential advantages over traditional methods (11).

The RLB, introduced in 2006, was developed as a simpler alternative to the thoracic paravertebral block (TPVB) (12). Unlike TPVB, which requires piercing the superior costotransverse ligament, the RLB targets the bony vertebral lamina, reducing invasiveness (13). Local anesthetic (LA) is injected into the fascial plane between the posterior thoracic lamina and the overlying transversus spinae muscles (12). Ultrasound guidance allows direct visualization of the lamina, muscle, and LA spread, improving precision (14). Recent studies, including cadaveric models, confirm that LA injected during a RLB extends through the intertransverse ligaments into the paravertebral and epidural spaces, covering 2 - 4 segmental levels (15, 16). A 2022 clinical trial demonstrated that ultrasound-guided RLBs significantly reduced opioid consumption in patients undergoing laparoscopic cholecystectomy compared to conventional analgesia (17). These findings highlight the RLB’s potential as a safe and effective option for postoperative pain management.

The ESPB, first described in 2021, is an ultrasound-guided technique involving LA injection into the fascial plane between the transverse thoracic processes and the erector spinae muscle, specifically the longissimus thoracis (18). Conceptually similar to the RLB, the ESPB differs in its bony landmark, targeting the transverse processes (19). Cadaveric and imaging studies show that LA spreads into the paravertebral and epidural spaces, as well as the lateral cutaneous branches of the intercostal nerves, covering 3 - 5 segmental levels in the epidural space and 6 - 10 levels in the intercostal region (20, 21). A meta-analysis reported that ESPB provided superior analgesia and reduced opioid requirements compared to TPVB in upper abdominal surgeries (22). Additionally, a randomized controlled trial showed that ESPB improved postoperative respiratory function and shortened recovery time in laparoscopic cholecystectomy patients (23). These findings underscore the ESPB’s broad analgesic coverage and its potential as a leading technique for postoperative pain management.

## 2. Objectives

Clinical case reports have demonstrated the efficacy of both RLB and ESPBs. Given these promising findings, our study aims to evaluate the use of these methods in adult laparoscopic surgery. Specifically, we will conduct a clinical trial to assess the impact of pre-emptive ropivacaine injection on postoperative pain management. This objective aligns with our broader goal of enhancing patient comfort and recovery outcomes.

## 3. Methods

### 3.1. Study Design 

**Figure 1.**
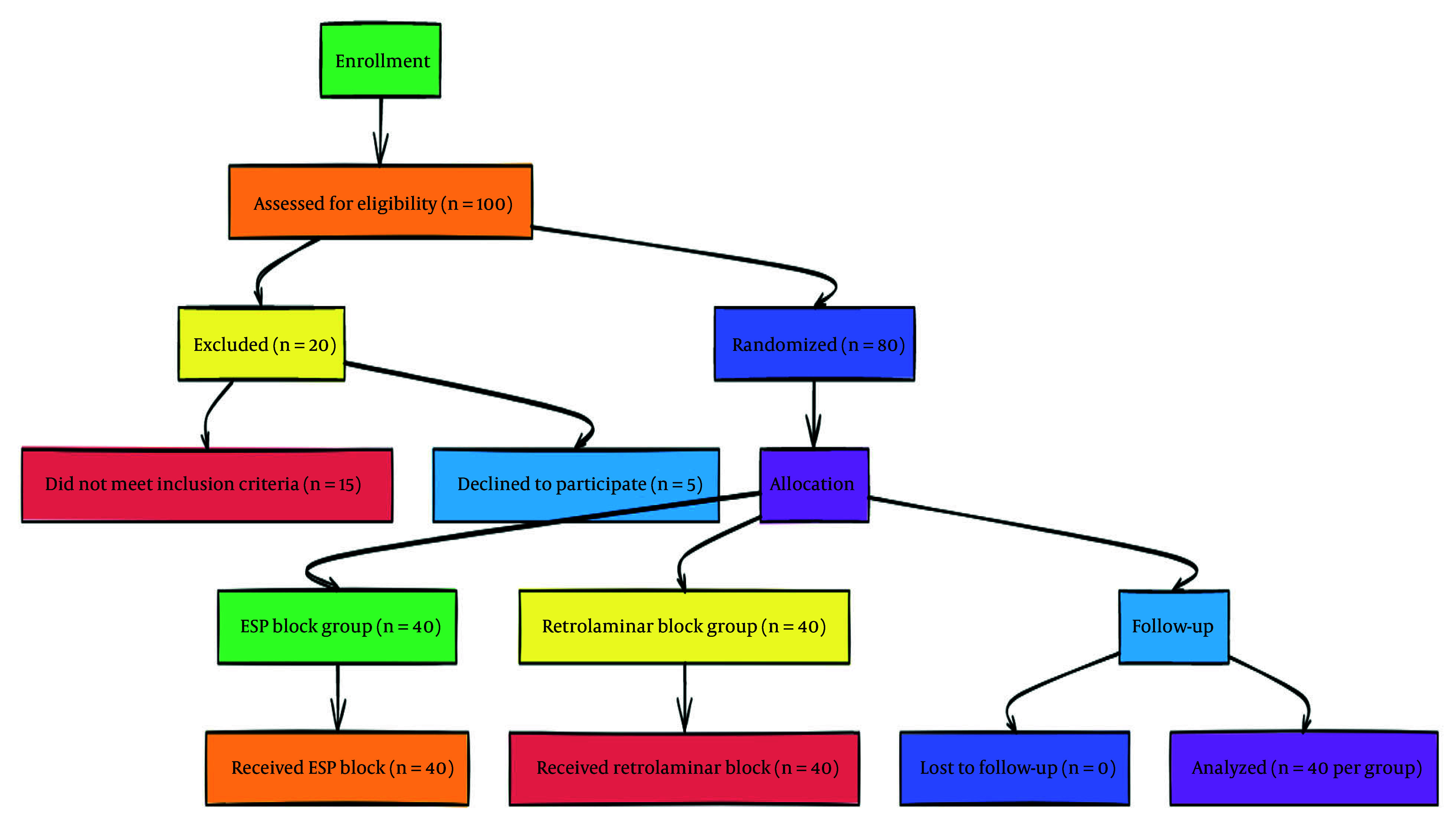
CONSORT diagram

The present study is a double-blind, randomized controlled trial conducted at Hazrat Rasool Akram Hospital and Firozgar Hospital, adhering to the Consolidated Standards of Reporting Trials (CONSORT) guidelines (24). A CONSORT flow diagram is provided to illustrate participant flow through the study (Figure 1). 

### 3.2. Eligibility Criteria

Patients aged 20 - 60 years with an American Society of Anesthesiologists (ASA) physical status classification of ≤ 2, undergoing elective upper abdominal laparoscopic surgeries (e.g., laparoscopic cholecystectomy for acute cholecystitis or gallstones), and providing written informed consent are eligible. Exclusion criteria include emergency surgery, organic comorbidities, hypersensitivity to ropivacaine, analgesic use within 24 hours pre-surgery, BMI > 35, liver or kidney disease, coagulopathy, opioid addiction, conversion to open surgery, or intraoperative complications.

### 3.3. Sample Size Calculation

The sample size was calculated to detect a clinically significant difference of 1.5 points in the Numeric Rating Scale (NRS) score between groups, with a standard deviation of 2.0 based on prior studies (25). Using a two-sided *t*-test with 80% power and a 5% significance level, a minimum of 36 patients per group was required. Accounting for a 10% dropout rate, 40 patients per group (total n = 80) were enrolled.

### 3.4. Randomization and Blinding

Patients were randomized into two groups — ESPB or RLB — using a computer-generated random sequence (random permuted blocks of size 4) to ensure balanced allocation. The randomization sequence was concealed in sealed, opaque envelopes opened only at the time of intervention. Double-blinding was achieved as follows: Patients were unaware of their group assignment, and the outcome assessor (a nurse who collected NRS scores and secondary outcomes) was blinded to the intervention. The anesthesiologist who performed the block was not involved in outcome assessment to maintain blinding.

### 3.5. Standards of Care

All patients underwent standard monitoring in the operating room, including pulse oximetry, non-invasive blood pressure, electrocardiogram, and Bispectral Index (BIS) or Analgesia Nociception Index (ANI). General anesthesia was administered uniformly: Premedication with fentanyl (2 µg/kg) and midazolam (0.12 mg/kg), induction with propofol (2 mg/kg) and cis-atracurium (0.2 mg/kg), and maintenance with propofol (100 µg/kg/min) and cis-atracurium (2 mg every 30 minutes). Fentanyl was titrated intraoperatively based on the ANI scale, with the total dose recorded at the end of surgery. Fluid and blood replacement followed standard protocols. Postoperatively, a patient-controlled intravenous analgesia (PCIA) pump containing 1200 µg of fentanyl in 100 mL saline was provided. The pump was set to deliver a 20 µg bolus (2 mL) per patient request, with a lockout interval of 15 minutes and a maximum of 80 µg per hour. Patients were instructed to record the time of their first analgesic request, and the number of boluses was logged at each follow-up visit.

### 3.6. Study Interventions

#### 3.6.1. Erector Spinae Plane Block

Patients were positioned laterally, and a curved ultrasound probe was aligned longitudinally at the T7 vertebra on the surgical side. After the transverse processes were identified, a needle was inserted under ultrasound guidance into the fascial plane beneath the erector spinae muscle. A 20 mL dose of 0.1% ropivacaine was injected, with spread confirmed by ultrasound.

#### 3.6.2. Retrolaminar Block 

Patients were positioned laterally, and a curved ultrasound probe was aligned longitudinally at the T7 vertebra. The vertebral lamina and spinous process were identified. A needle was inserted 1 - 1.5 cm lateral to the spinous process, advanced toward the lamina, and 20 mL of 0.1% ropivacaine was injected into the fascial plane between the lamina and transversus spinae muscles. All blocks were performed by an anesthesiologist experienced in ultrasound-guided techniques.

### 3.7. Outcomes Assessment

#### 3.7.1. Primary Outcome 

The primary outcome was postoperative analgesia, measured by the NRS (0 - 10) at rest and during coughing at 0, 2, 4, 6, 12, and 24 hours post-surgery.

#### 3.7.2. Secondary Outcomes

(1) Narcotic consumption: Total fentanyl used via PCIA in the first 24 hours, recorded as micrograms.

(2) Time to first analgesic request: Time from surgery completion to the first PCIA bolus.

(3) Nausea and vomiting: Assessed using a binary scale (present/absent) at the same time points as NRS, based on patient self-report or clinical observation (e.g., emesis or antiemetic use).

(4) Patient satisfaction: Measured 24 hours post-surgery using a 5-point scale (0 = poor, 4 = excellent).

(5) Complications: Any adverse events during blocks (e.g., bleeding, hypotension, infection) are documented.

### 3.8. Statistical Analysis

Data were analyzed using SPSS version 26. Continuous variables (e.g., NRS scores, fentanyl consumption) were compared using independent *t*-tests or Mann-Whitney U tests, depending on normality (assessed via Shapiro-Wilk test). Categorical variables (e.g., nausea incidence) were analyzed using chi-square or Fisher’s exact tests. Repeated-measures ANOVA was used to evaluate NRS scores over time. A P-value < 0.05 was considered statistically significant. Missing data were handled using last-observation-carried-forward imputation.

### 3.9. Ethical Considerations

The study adheres to the Declaration of Helsinki and is approved by the Ethics Committee of Iran University of Medical Sciences (IR.IUMS.REC.1402.123). Participation is voluntary, with no additional costs to patients. Questionnaires are anonymous, and data are reported in aggregate to ensure confidentiality.

## 4. Results

The present study involved 80 patients who were referred to Hazrat Rasool and Firozgar hospitals for upper abdominal laparoscopic surgeries. These patients were randomly allocated into 2 groups: The ESPB group (n = 40) and the RLB group (n = 40). The mean age of the patients was 48 ± 8.44 years. Of these patients, 65% (n = 52) were female and 35% (n = 28) were male. The mean Body Mass Index (BMI) of the patients was 26.7 ± 6.51. An investigation of the demographic and baseline characteristics in the 2 intervention groups revealed no statistically significant differences (P > 0.05). Table 1 presents the demographic and baseline characteristics of the patients in the ESPB and RLB groups.

**Table 1.** Demographic and Baseline Characteristics of Patients in the Erector Spinae Plain Block and Retrolaminar Block Groups ^a^

Variables	Blocks		P-Value ^d^
	ESPB ^b^	RLB ^c^	
**Age**	47.27 ± 6.72	48.92 ± 9.88	0.385
**Gender**			1.000
Female	26 (50.0)	26 (50.0)	
Male	14 (50.0)	14 (50.0)	
**ASA ** ^**e**^			0.370
No	19 (44.2)	24 (55.8)	
Yes	21 (56.8)	16 (43.2)	
**BMI ** ^**f**^	27.66 ± 8.98	25.76 ± 1.84	0.420
**Time (surgery)**	2.27 ± 0.33	2.17 ± 0.31	0.179

^a^ Data are presented as No. (%) or mean ± SD.

^b^ ESPB: Erector spinae plane block, an ultrasound-guided regional anesthesia technique involving injection of LA into the fascial plane beneath the erector spinae muscle.

^c^ RLB: Retrolaminar block, an ultrasound-guided regional anesthesia technique involving injection of LA into the fascial plane between the vertebral lamina and transversus spinae muscles.

^d^ A P-value of < 0.05 is considered statistically significant.

^e^ ASA: American Society of Anesthesiologists physical status classification, where ASA I indicates a healthy patient and ASA II indicates a patient with mild systemic disease.

^f^ BMI: Body Mass Index, calculated as weight in kilograms divided by the square of height in meters (kg/m^2^).

The average NRS scores at various time points (0, 20 minutes, 2 hours, 4 hours, 6 hours, 12 hours, and 24 hours) post-operation were evaluated using a repeated measures test. The results indicated statistically significant intra-group changes in the average NRS scores at these time points in both the ESPB and RLB intervention groups (P < 0.001). Furthermore, the average NRS scores in the ESB group were significantly higher than those in the RL group at all time points post-operation, excluding the initial time point (P < 0.05). Table 2 presents the mean and standard deviation of the NRS scores at the specified time points post-surgery for both the ESB and RL intervention groups.

**Table 2.** Mean and Standard Deviation of Numeric Rating Scale Scores at Various Time Points Post-operation in the Erector Spinae Plane Block and Retrolaminar Block Groups ^a^

Variables and Blocks	No.	Mean ± SD	Mean Difference (95% CI) ^b^	P-Value ^c^
**NRS ** ^**d**^ ** (0)**			0.0250 (-0.28725 - 0.3372)	0.568
RLB ^e^	40	4.007 ± 0.766		
ESBP ^f^	40	4.100 ± 0.671		
**NRS (20 min)**			1.3500 (1.1100 - 1.5893)	< 0.001
RLB	40	1.650 ± 0.833		
ESBP	40	2.925 ± 0.416		
**NRS (2 h)**			1.0250 (0.89773 - 1.1522)	< 0.001
RLB	40	1.100 ± 0.303		
ESBP	40	2.100 ± 0.303		
**NRS (4 h)**			1.1500 (1.0047 - 1.2952)	< 0.001
RLB	40	1.050 ± 0.220		
ESBP	40	2.200 ± 0.405		
**NRS (6 h)**			1.4500 (1.1477 - 1.7522)	< 0.001
RLB	40	1.775 ± 0.767		
ESBP	40	3.225 ± 0.576		
**NRS (12 h)**			0.8000 (0.5459 - 1.0540)	< 0.001
RLB	40	2.400 ± 0.632		
ESBP	40	3.250 ± 0.543		
**NRS (24 h)**			0.2750 (0.0673 - 0.4826)	0.010
RLBP	40	2.100 ± 0.378		

^a^ Data are presented as mean ± standard deviation for NRS scores, with mean differences and 95% confidence intervals calculated to compare groups.

^b^ Mean Difference (95% CI): Difference in mean NRS scores between RLB and ESPB groups, with 95% confidence interval.

^c^ A P-value of < 0.05 is considered statistically significant.

^d^ NRS: Numeric Rating Scale, a pain intensity scale ranging from 0 (no pain) to 10 (worst possible pain), measured at rest at specified time points post-operation.

^e^ RLB: retrolaminar block, an ultrasound-guided regional anesthesia technique involving injection of LA into the fascial plane between the vertebral lamina and transversus spinae muscles.

^f^ ESPB: Erector spinae plane block, an ultrasound-guided regional anesthesia technique involving injection of LA into the fascial plane beneath the erector spinae muscle.

**Figure 2.**
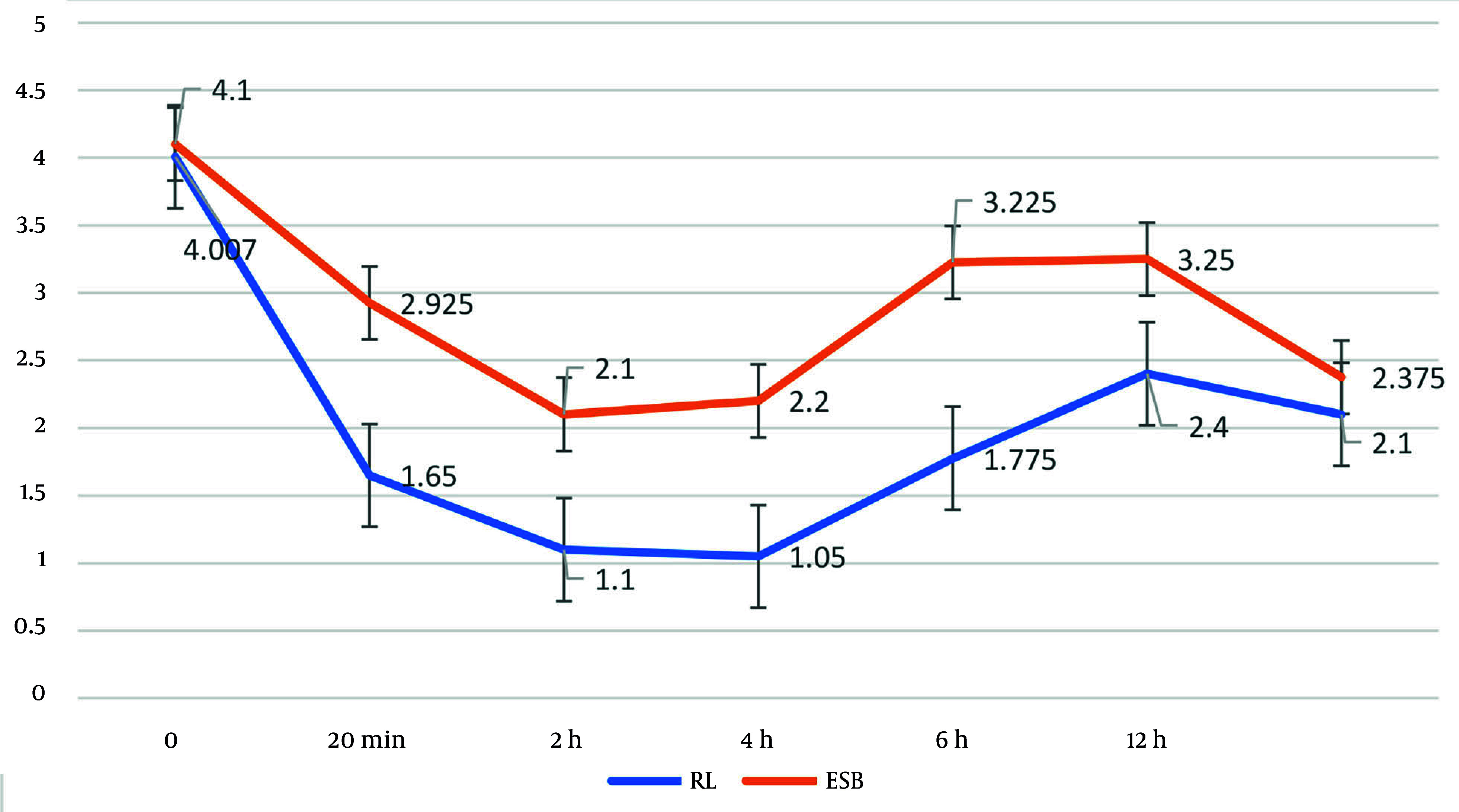
Changes in Numeric Rating Scale (NRS) score at various time points post-operation in the erector spinae plane block (ESPB) and retrolaminar block (RLB) groups

Patient satisfaction levels at various time points (0, 20 minutes, 2 hours, 4 hours, 6 hours, 12 hours, and 24 hours) post-operation were assessed in the ESPB and RLB intervention groups using the Friedman test. The results indicated statistically significant changes in patient satisfaction levels at these time points in both intervention groups (P < 0.001). A comparison of patient satisfaction levels at each time point between the two blocks revealed that the satisfaction levels at 20 minutes, 2 hours, 4 hours, and 6 hours post-operation were significantly different (P < 0.05). However, there was no statistically significant difference at other time points between the two intervention groups (P > 0.05) (Figure 2). Table 3 presents the patient satisfaction levels at the specified time points post-operation for both the ESPB and RLB intervention groups.

**Table 3.** Patient Satisfaction Levels at Various Time Points Post-operation in the Erector Spinae Plane Block and Retrolaminar Block Groups ^a^

Variables	Blocks		P-Value ^d^
	ESBP ^b^	RLB ^c^	
**Patient satisfaction ** ^**e**^ ** (0)**			0.618
Excellent	4 (10.0)	4 (10.0)	
Very good	25 (62.5)	21 (52.5)	
Moderate	11 (27.5)	15 (37.5)	
**Patient satisfaction (20 min)**			0.001
Excellent	16 (40.0)	32 (80.0)	
Very good	23 (57.5)	7 (17.5)	
Moderate	1 (2.5)	1 (2.5)	
**Patient satisfaction (2 h)**			0.152
Excellent	38 (95.0)	40 (100.0)	
Very good	2 (5.0)	0 (0.0)	
**Patient satisfaction (4 h)**			0.077
Excellent	37 (92.5)	40 (100.0)	
Very good	3 (7.5)	0 (0.0)	
**Patient satisfaction (6 h)**			0.002
Excellent	19 (47.5)	32 (80.0)	
Very good	21 (52.5)	8 (20.0)	
**Patient satisfaction (12 h)**			0.348
Excellent	24 (60.0)	28 (70.0)	
Very good	16 (40.0)	12 (30.0)	
**Patient satisfaction (24 h)**			1.000
Excellent	36 (90.0)	36 (90.0)	
Very good	4 (10.0)	4 (10.0)	

^a^ Data are presented as No. (%) of patients in each satisfaction category, with comparisons between groups analyzed using chi-square or Fisher’s exact tests.

^b^ ESPB: Erector spinae plane block, an ultrasound-guided regional anesthesia technique involving injection of LA into the fascial plane beneath the erector spinae muscle.

^c^ RLB: Retrolaminar block, an ultrasound-guided regional anesthesia technique involving injection of LA into the fascial plane between the vertebral lamina and transversus spinae muscles.

^d^ A P-value of < 0.05 is considered statistically significant.

^e^ Patient satisfaction: Measured on a categorical scale (excellent, very good, moderate) at specified time points post-operation, reflecting patients’ subjective experience of pain relief and comfort.

The time of the first narcotic request and the total narcotic dose consumed in the 24 hours post-operation were examined in both the ESPB and RLB intervention groups using an independent *t*-test. The results revealed that the total narcotic dose consumed in the 24 hours post-operation was significantly higher in the ESPB group (P = 0.050). However, the average time of the first narcotic request did not significantly differ between the two groups (P = 0.669). Table 4 presents the mean and standard deviation of the time of the first narcotic request and the total narcotic dose consumed in the 24 hours post-operation for both the ESPB and RLB intervention groups.

**Table 4.** Mean and Standard Deviation of Time of First Narcotic Request and Total Narcotic Dose Consumed in 24 Hours Post-operation in the Erector Spinae Plane Block and Retrolaminar Block Groups ^a^

Variables and Blocks	No.	Mean ± SD	Mean Difference (95% CI) ^b^	P-Value ^c^
**First narcotic request ** ^**d**^			0.060 (-0.2184 - 0.3384)	0.669
ESBP ^e^	40	5.502 ± 0.639		
RLB ^f^	40	5.442 ± 0.611		
**Total narcotic dose consumed in the 24 hours ** ^**g**^ ** (mg)**			3.750 (0.0161 - 7.4839)	0.050
ESBP	40	6.250 ± 9.789		
RLB	40	2.500 ± 6.698		

^a^ Data are presented as mean ± standard deviation, with comparisons between groups analyzed using independent *t*-tests or Mann-Whitney U tests, depending on data normality.

^b^ Mean difference (95% CI): Difference in mean values between ESPB and RLB groups, with 95% confidence interval.

^c^ A P-value of < 0.05 is considered statistically significant.

^d^ First narcotic request: Time (in hours) from surgery completion to the first patient-initiated request for narcotic analgesia via PCIA.

^e^ ESPB: Erector spinae plane block, an ultrasound-guided regional anesthesia technique involving injection of LA into the fascial plane beneath the Erector Spinae muscle.

^f^ RLB: Retrolaminar block, an ultrasound-guided regional anesthesia technique involving injection of LA into the fascial plane between the vertebral lamina and transversus spinae muscles.

^g^ Total narcotic dose consumed: Total amount of fentanyl (in micrograms, µg) administered via PCIA in the first 24 hours post-operation.

## 5. Discussion

The findings of this double-blind randomized controlled trial comparing the ESPB and the RLB provide valuable insights into their efficacy for postoperative pain management following laparoscopic upper abdominal surgeries. The RLB demonstrated superior pain control, as evidenced by significantly lower NRS scores at most time points post-operation (20 minutes, 2, 4, 6, 12, and 24 hours) compared to the ESPB, except at time zero. Additionally, patient satisfaction was higher in the RLB group at 20 minutes, 2 hours, 4 hours, and 6 hours, and the total narcotic dose consumed over 24 hours was significantly lower in the RLB group, suggesting a reduced reliance on opioids.

These results align with prior research. For instance, Liu et al. reported lower pain scores and reduced inflammatory markers with RLB compared to other regional blocks, although they used the Visual Analog Scale (VAS) rather than NRS (26). Similarly, Zhao et al. found that RLB was associated with lower VAS scores, reduced morphine consumption, and improved postoperative respiratory function compared to ESPB (27). The lower narcotic consumption observed in the RLB group in our study corroborates these findings, highlighting RLB’s potential to enhance recovery by minimizing opioid-related side effects, such as PONV.

However, our study found no significant difference in the time to first narcotic request or PONV incidence between groups, consistent with Sotome et al.’s findings in breast cancer surgery patients, where no differences were noted in pain medication request timing or PONV rates (28). The superior performance of RLB may be attributed to its anatomical targeting. The RLB delivers LA into the fascial plane between the vertebral lamina and transversus spinae muscles, potentially achieving more consistent spread to the paravertebral and epidural spaces (14). In contrast, the ESPB targets the fascial plane beneath the erector spinae muscle, which may result in a broader but less predictable spread (27). These anatomical differences could explain the observed differences in pain control and narcotic requirements.

The generalizability of these findings is limited by the study’s focus on patients undergoing elective laparoscopic upper abdominal surgeries, specifically cholecystectomy, with ASA status I or II and BMI < 35. The results may not directly apply to patients with higher ASA classifications, obesity, or those undergoing other surgical procedures, such as open abdominal surgeries or thoracic procedures, where anatomical and pain characteristics differ. Additionally, the study was conducted in a specific geographic and clinical setting (two hospitals in Iran), which may limit applicability to diverse populations with varying healthcare practices or pain perception thresholds. Future studies should explore RLB and ESPB efficacy in broader surgical contexts, including non-laparoscopic procedures and diverse patient demographics, to enhance generalizability.

### 5.1. Conclusions

This study provides evidence that both ESPB and RLB are effective for postoperative pain management in laparoscopic upper abdominal surgeries, with RLB demonstrating superior pain control and reduced narcotic consumption. These findings contribute to the growing body of evidence supporting ultrasound-guided regional anesthesia and highlight RLB as a valuable technique for improving patient outcomes. Continued research will further elucidate the optimal applications of these blocks across diverse clinical scenarios.

### 5.2. Limitations

Several limitations should be acknowledged. First, inter-operator variability in block administration may have influenced outcomes, as the success of ultrasound-guided blocks depends on the skill and experience of the anesthesiologist. Although all blocks were performed by a skilled practitioner, subtle differences in technique could affect LA spread and efficacy. Second, the study was conducted across two hospitals in a single geographic region, which may limit the external validity of the findings compared to multicenter trials that capture greater variability in clinical practice. Third, the sample size (n = 80, 40 per group) was sufficient to detect a clinically significant difference in NRS scores but may have been underpowered to detect differences in secondary outcomes, such as PONV incidence or time to first narcotic request. Larger studies could provide more robust evidence of these outcomes. Finally, the study did not assess long-term outcomes, such as chronic pain development, which could further elucidate the comparative benefits of RLB and ESPB.

### 5.3. Implications and Future Directions

The RLB’s ability to provide effective analgesia with reduced narcotic use positions it as a promising technique for multimodal pain management strategies. Its lower opioid requirement may reduce the risk of opioid-related complications, supporting enhanced recovery protocols. However, the lack of significant differences in PONV and time to first narcotic request suggests that both blocks offer comparable early postoperative outcomes in some aspects. Future research should address the identified limitations by conducting multicenter trials with larger sample sizes to confirm these findings and enhance generalizability. Additionally, studies should investigate the efficacy of RLB and ESPB in other surgical populations, such as pediatric or elderly patients, or in procedures like thoracic or orthopedic surgeries. Exploring standardized training protocols to minimize inter-operator variability and evaluating long-term outcomes, such as chronic pain, will further optimize the application of these techniques.

## Data Availability

The dataset presented in the study is available on request from the corresponding author during submission or after publication. The data are not publicly available due to privacy and ethical considerations, as the dataset contains sensitive patient information that cannot be shared without explicit consent from the participants.

## Clinical Trial Registration Code:

IRCT20120814010599N28.
